# Detrended Fluctuation, Coherence, and Spectral Power Analysis of Activation Rearrangement in EEG Dynamics During Cognitive Workload

**DOI:** 10.3389/fnhum.2019.00270

**Published:** 2019-08-08

**Authors:** Ivan Seleznov, Igor Zyma, Ken Kiyono, Sergii Tukaev, Anton Popov, Mariia Chernykh, Oleksii Shpenkov

**Affiliations:** ^1^Department of Electronic Engineering, National Technical University of Ukraine “Igor Sikorsky Kyiv Polytechnic Institute”, Kyiv, Ukraine; ^2^Department of Physiology and Anatomy, Educational and Scientific Center “Institute of Biology and Medicine”, National Taras Shevchenko University of Kyiv, Kyiv, Ukraine; ^3^Division of Bioengineering, Graduate School of Engineering Science, Osaka University, Osaka, Japan; ^4^Department of Physiology of Brain and Psychophysiology, Educational and Scientific Centre “Institute of Biology and Medicine”, National Taras Shevchenko University of Kyiv, Kyiv, Ukraine; ^5^Department of Social Communication, Institute of Journalism, National Taras Shevchenko University of Kyiv, Kyiv, Ukraine; ^6^Laboratory on Theory and Methodic of Sport Preparation and Reserve Capabilities of Athletes, Scientific Research Institute, National University of Physical Education and Sports of Ukraine, Kyiv, Ukraine; ^7^R&D Engineering, Ciklum, London, United Kingdom; ^8^Department of Biophysics and Medical Informatics, Educational and Scientific Center “Institute of Biology and Medicine”, Taras Shevchenko National University of Kyiv, Kyiv, Ukraine

**Keywords:** electroencephalogram, detrended fluctuation analysis, cognitive workload, brain dynamics, coherence, power spectral density

## Abstract

In the study of human cognitive activity using electroencephalogram (EEG), the brain dynamics parameters and characteristics play a crucial role. They allow to investigate the changes in functionality depending on the environment and task performance process, and also to access the intensity of the brain activity in various locations of the cortex and its dependencies. Usually, the dynamics of activation of different brain areas during the cognitive tasks are being studied by spectral analysis based on power spectral density (PSD) estimation, and coherence analysis, which are *de facto* standard tools in quantitative characterization of brain activity. PSD and coherence reflect the strength of oscillations and similarity of the emergence of these oscillations in the brain, respectively, while the concept of stability of brain activity over time is not well defined and less formalized. We propose to employ the detrended fluctuation analysis (DFA) as a measure of the EEG persistence over time, and use the DFA scaling exponent as its quantitative characteristics. We applied DFA to the study of the changes in activation in brain dynamics during mental calculations and united it with PSD and coherence estimation. In the experiment, EEGs during resting state and mental serial subtraction from 36 subjects were recorded and analyzed in four frequency ranges: θ1 (4.1–5.8 Hz), θ2 (5.9–7.4 Hz), β1 (13–19.9 Hz), and β2 (20–25 Hz). PSD maps to access the intensity of cortex activation and coherence to quantify the connections between different brain areas were calculated, the distribution of DFA scaling exponent over the head surface was exploited to measure the time characteristics of the dynamics of brain activity. Obtained arrangements of DFA scaling exponent suggest that normal functioning of the brain is characterized by long-term temporal correlations in the cortex. Topographical distribution of the DFA scaling exponent was comparable for θ and β frequency bands, demonstrating the largest values of DFA scaling exponent during cognitive activation. The study shows that the long-term temporal correlations evaluated by DFA can be of great interest for diagnosis of the variety of brain dysfunctions of different etiology in the future.

## Introduction

The study of the human cognitive activity is an active field of theoretical and practical investigations (Sarter et al., 1996; Baars, 2005; Eysenck and Brysbaert, 2018; Illeris, 2018). Among other topics, the study of the brain dynamics during the cognition process has attracted a lot of attention from the researchers (Bressler and Kelso, 2001; Beaty et al., 2016). The dynamics of activation of different brain areas during the cognitive tasks has been mainly studied by the spectral analysis based on Fast Fourier Transform, and coherence analysis (Kropotov, 2010). These two well-established methods provide a powerful tool which complimentary access the brain activation dynamics and allowed to reveal important patterns of brain activity during various experimental settings.

Traditionally, a significant part of electroencephalogram (EEG) studies use Power Spectral Density (PSD) to find the key discriminative emotional features related to the arousal and valence in response to videos (Soleymani et al., 2012), while listening to the music (Lin et al., 2010), to detect features that are associated with induced pleasant emotion (Kortelainen et al., 2015), to create the informative classification of emotions based on the changes in the gamma band, and to find the most informative EEG channels for emotion recognition (Li and Lu, 2009). In Kortelainen and Seppänen (2013), the evoked response synchronization/desynchronization during emotions in different frequency bands by using the Fourier spectral analysis was examined.

On the other hand, coherence reflects the distant synchronization of the neural oscillations in different brain regions related to functional connectivity, which is also possible to access in every frequency range of interest. It allows studying the connections between functional and morphological brain areas during different tasks realization, such as language (Weiss and Mueller, 2003) and numerical magnitude processing (González-Garrido et al., 2018) etc.

Cognitive activation is a complex time-space process in the brain, for which PSD and coherence jointly reflect the strength of oscillations and similarity of the emergence of these oscillations in the brain. But, to our knowledge, one important feature of the oscillations is not very well defined and used yet, which is stability of the oscillatory process on the brain over time. We suppose that it would be beneficial to quantify the persistence and stability of the oscillatory activity over time in each region of the brain, to highlight the duration of the brain electrical activity periods with stable characteristics and study the dynamics of the rearrangement of the activations for different regions. In this work, we propose to consider the Detrended Fluctuation Analysis (DFA) as a tool to define such stability. DFA is the method of studying the non-linear characteristics of signal which quantifies the presence of long- and short-term correlations in time series.

DFA was rarely used before as a complementary to the standard techniques in the analysis of cognitive processes. DFA was applied to quantify the complexity of alpha and theta EEG rhythms during listening to meditation music (Maity et al., 2015). It was revealed that the complexity of alpha and theta rhythms in the form of multifractal spectral width increases in seven frontal locations (F3, F4, F7, F8, Fp1, Fp2, and Fz) under the effect of musical stimuli. DFA was used to analyze EEG characteristics during three emotional states induced by music listening (fear, happiness, sadness) and under the rest condition with eyes-closed (Gao et al., 2007). Two scaling regions were identified in a log-log plots, with empirically estimates bending point corresponding to the alpha frequency range (8–12 Hz). ANOVA results showed that the DFA scaling exponents in two scaling regions provide a simple summary of the complex dynamics in EEG during listening to emotional music.

In the study of the emotional characteristics of EEG by using DFA and Power Spectral Intensity (Sanyal et al., 2015), the correlation was studied between the variation in scaling exponent and the changes in spectral power in the alpha and delta ranges while listening to the contrasting music (eliciting two contrasting emotions, happiness and sorrow) vs. the resting state. The most prominent result was the fact that alpha power decreased during stimuli, while complexity either increases or decreases depending on the music type. In Banerjee et al. (2016), the Fast Fourier Transform and DFA is used to analyze the EEG (frontal areas, F3–F4) recorded while listening to music at three experimental conditions (rest state, listening to the music and without music). Frequency analysis was performed for the alpha, theta and gamma brain rhythms. In the same time, DFA of the alpha rhythm shows a retention of the emotion even after the withdrawal of both happy and sad music. Also, this work observed the residual arousals reflected in both alpha rhythm and DFA exponent after the musical stimulus were removed, which is similar to the conventional “Hysteresis” loop. This finding evokes the new potential field of study of time dynamics of response to emotional stimuli.

To our knowledge, none of the previous works was focused on the systematic study of DFA and its comparison to well-established techniques, especially coherence. Therefore, DFA needs to be studied more with respect to its potential application to the research in cognitive neuroscience. In this work, we aim to study the DFA characteristics of the neuronal dynamics and emotional and cognitive characteristics of the brain during cognitive workload in comparison to standard techniques of PSD and coherence. Our hypothesis is that DFA can provide complementary information about the stability of the neuronal oscillations in the time domain, in addition to the strength of such oscillations (from PSD) and synchronization of activity in different regions (from coherence).

To demonstrate this approach, we have chosen mental arithmetic task performance (serial subtraction) as a model of activation mechanism during the execution of external cognitive task. The mental calculation is a traditionally used method of inducing the cognitive load and there are different versions of proposed count schemes: the serial calculation (subtraction, addition, multiplication, division) and the selection the correct sum on the screen (Burbaud et al., 1995; Stam et al., 1996; Dehaene et al., 1999; Menon et al., 2000; Pinheiro-Chagas et al., 2019). The use of classical neurophysiological methods, electroencephalography, magnetoencephalography (MEG), functional magnetic resonance imaging (fMRI) revealed the neuronal organization, the brain structures involved in this type of cognitive activity. The tripartite organization of calculation was revealed in Dehaene et al. (2003): the horizontal segment of the intraparietal sulcus is the specific domain for the semantic representation of numbers. The activation of the left angular gyrus area in connection with other left-hemispheric perisylvian areas indicates that the manipulation of numbers happens in verbal form. In Dehaene et al. (2004) and Kong et al. (2005), the activation of the intraparietal sulcus in arithmetic tasks realization such as the subtraction has been shown. The activation of the two main components of the calculation circuits (the left inferior frontal area for exact calculation and the bilateral intraparietal area for approximation) was detected approximately at 230 and 280 ms poststimulus (Dehaene et al., 1999).

During mental calculation, the activation in the dorsolateral prefrontal cortex (the cortical surface or in the anterior bank of the left gyrus frontalis medius—Brodmann’s area 46), in the left insula and the cingulate or temporal cortex (rarely) was observed (Burbaud et al., 1995). Authors of Menon et al. (2000) found the effect of arithmetic complexity on the recruitment of the left and right angular gyrus. The dissociation in prefrontal and parietal cortex function happened during arithmetic processing. The studies (Asada et al., 1999; Enriquez-Geppert et al., 2014) have demonstrated that the dorsal part of the anterior cingulate cortex and the adjacent medial the prefrontal cortex are responsible for the cognitive functions connected with mental arithmetic. In these areas, θ-rhythm generators of frontal midline are localized. A significant increase in the power of the Frontal Midline θ is detected with the complexity, difficulty of the task. Also, frontal brain areas (Fθ) are leaders in the relationship with parieto-occipital (POα2) brain regions under cognitive load and these causal interactions are enhanced with absolute accuracy. The failure of mental arithmetic is reflected in the zero time-lag between bilateral frontal θ and α2 in the right parieto-occipital area (Dimitriadis et al., 2016).

The analysis of the aforementioned up-to-date state in studies of emotional activation allows making several conclusions:

- All studies demonstrate strong quantitative variations of response for the emotional stimuli or cognitive load in terms of oscillatory power while mostly frontal areas are studied,
- Standard EEG frequency ranges are mostly used without the specification for the frequency subbands which are more relevant to the specific state/function,
- Absence of studying the persistence of neuronal oscillations during mental cognitive tasks performance, which we hypothesize the DFA application can provide.

Considering all of the above, the aims of the present study includes revealing whether the application of DFA allows to investigate the formation of neural networks’ stable states during intensive mental activity, assessing the effectiveness of DFA in comparison with existing methods of functional connectivity and oscillatory power mapping, as well as highlighting specific emotional and cognitive features of the activation-related neurodynamics during mental arithmetic task performance.

## Materials and Methods

### Data Collection

The detailed description of the data used in the present study, experiment design, participants can be found in Zyma et al. (2019). Here, we briefly outline the main points.

### Participants

In total, 66 healthy right-handed volunteers (47 women and 19 men), 1st–3rd year students of the Taras Shevchenko National University of Kyiv (Educational and Scientific Centre “Institute of Biology and Medicine” and Faculty of Psychology) aged 18–26 years (M_age_ = 18.6, SD = 0.87 years) participated in this study. The participants were eligible to enroll in the study if they had normal or corrected-to-normal visual acuity, normal color vision, had no clinical manifestations of mental or cognitive impairment, verbal or non-verbal learning disabilities. Exclusion criteria were: the use of psychoactive medication, drug or alcohol addiction and psychiatric or neurological complaints.

The study was approved by the Bioethics Commission of Educational and Scientific Centre “Institute of Biology and Medicine,” Taras Shevchenko National University of Kyiv and written informed consent was obtained from each subject in accordance with the World Medical Association (WMA) Declaration of Helsinki—ethical principles for the medical research involving human subjects (Helsinki, Finland, June 1964), the Declaration of Principles on Tolerance (28th session of the General Conference of UNESCO, Paris, November 16, 1995), the Convention for the protection of Human Rights and Dignity of the Human Being with regard to the Application of Biology and Medicine: Convention on Human Rights and Biomedicine (Oviedo, April 04, 1997).

### EEG Recordings and Preprocessing

The EEGs were recorded monopolarly using Neurocom EEG 23-channel system (XAI-MEDICA, Ukraine). The electrodes (silver/silver chloride) were placed on the scalp at symmetrical anterior frontal (Fp1, Fp2), frontal (F3, F4, Fz, F7, F8), central (C3, C4, Cz) parietal (P3, P4, Pz), occipital (O1, O2) and temporal (T3, T4, T5, T6) recording sites according to the International 10-20 scheme. All electrodes were referenced to the interconnected ear reference electrodes. The inter-electrode impedance levels were below 5 kΩ. The sample rate of all channels was 500 Hz. Thirty of the 66 participants were rejected due to poor task performance or due to poor EEG quality (excessive amount of artifacts), so the final sample size was 36 subjects.

The Python 3.6 programming language was used to implement all data analysis and visualization in this work.

### Experiment Design

In present experiments, we have studied EEG correlates of mental activity—using the intensive cognitive task (mental arithmetic task—serial subtraction). Arithmetic tasks in present study involved the serial subtraction of two numbers. Each trial started with the communication orally 4-digit (minuend) and 2-digit (subtrahend) numbers (e.g., 4,753 and 17, 3,141 and 42 etc.). Mental arithmetic performance is considered as standardized stress-inducing experimental protocol (Jatoi et al., 2014; Finlay et al., 2016). Serial subtraction during 15-min is considered as a psychosocial stress (Noto et al., 2005). In this way, our study design required from the subjects the intensive cognitive activity. Intensive mental load is accompanied by a change in the emotional background when the subject takes additional efforts to resolve tasks, so one can talk about evoked emotions in this case.

During EEG recording, the participants were sitting in a soundproof dark chamber, in a cosy armchair in a comfortable reclining position. Prior to the experiment participants were instructed to try to relax during the rest state and informed about the arithmetic task—participants were asked to count mentally without speaking and using finger movements, accurately, and quickly in the rhythm they had reached. After 3 min of adaptation to experimental conditions and registration of the EEG of the rest state with closed eyes (3 min) the participants performed a mental arithmetic task—serial subtraction during 4 min.

In this study, we analyzed EEG during the last minute of rest state and the first minute of the mental arithmetic, since the strategy of the task performance is being formed at the present time and the emotional state of the participants is changing considerably due to intellectual overload.

### Behavioral Data Analysis

After the finishing of the arithmetic task execution, the subject informed the result of subtraction. The number of operations and correctness were computed for each participant. For each participant, a mental arithmetic score was obtained by subtracting the last number reached from a 4-digit number. The task performance was accurate if the score was an exact multiple of a corresponding 2-digit subtrahend. We had compared the mentally calculated subtraction across subjects and concluded that the participant had successfully engaged in the task if their reported result did not differ by more than 20% from the correct value. A similar approach in assessing the quality of the task was applied in the study (Kissler et al., 2000).

One of our aims was to assess the ability of participants to proceed with the arithmetic task. In this study, we investigate how brain activation changes as the dependent of individual task difficulty. This issue has been left unexplored by studies. The increase in the rate of presentation numbers is used to investigate the task difficulty (Menon et al., 2000). Individual task difficulty for participants can be assessed by the number of operations performed in unit time and by the characteristics of proposed numbers. In this work, to identify the EEG features associated with the task difficulty for the participants we used the variation series (ranked series) of behavior data as the basis of the grouping. Based on the number of arithmetic operations per minute, we divided the sample (36 subjects) into two groups: the proposed task was a difficult task for one group of participants (group “B,” 12 subjects, M_number operations_ = 7, Standard Deviation (SD) = 3.6), the second group effectively managed with the task (group “G,” 24 subjects, M_number operations_ = 21, SD = 7.4).

### EEG Frequency Ranges

For effective estimation of the changes in EEG due to the cognitive activation, the following frequency subbands were chosen:

1. θ1 (4.1, 5.8) Hz
2. θ2 (5.9, 7.4) Hz
3. β1 (13, 19.9) Hz
4. β2 (20, 25) Hz

Theta- and beta-band oscillations directly reflect such cognitive processes as retrieval and actualization of memory (Osipova et al., 2006; Bastiaansen et al., 2008), emotional excitement (Demiralp and Başar, 1992) and other consciousness-driven processes (Gundel and Wilson, 1992). Also, it has been shown the increased synchronization in theta-band particularly in its low-frequency subband during the execution of complex mental tasks, activation of the processes underlying working memory, as well as in neurodynamics in altered states of consciousness (Molnár et al., 2005). The reasoning behind the focusing only on these frequency ranges will be provided in the “Discussion” section.

To establish EEG correlates of emotions (emotion features) during mental load and during rest state, we conducted DFA, Oscillatory Power (PSD) and coherence analysis (Analysis of Functional Connectivity) of the recorded data.

### Detrended Fluctuation Analysis

**Figure 1:**
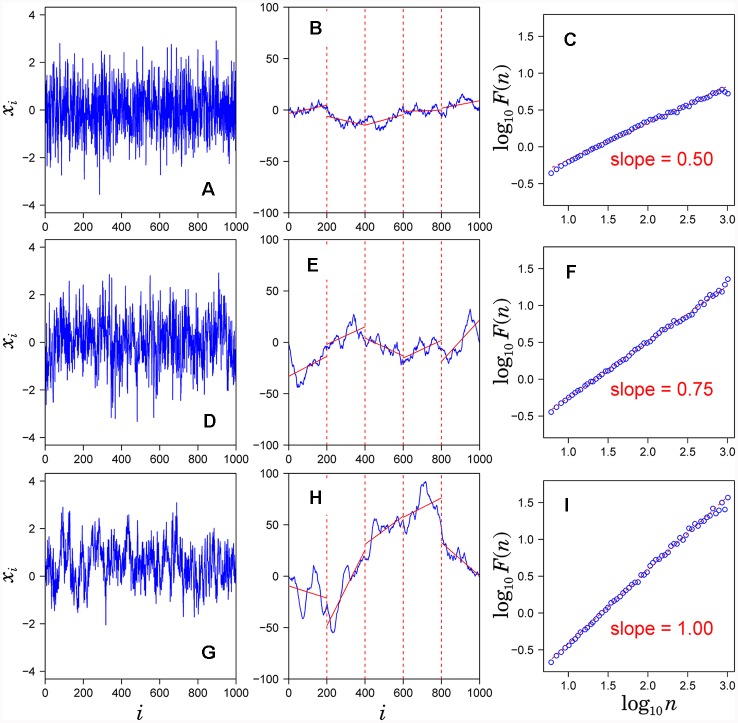
Assessment of scaling exponents of time series {xi} by detrended fluctuation analysis (DFA). **(A,D,G)** Examples of time series. **(B,E,H)** Integrated series (blue solid lines) of the time series shown in the left hand panel. **(C,F,I)** Log-log plot of F(*n*) vs. *n*. The scaling exponent α is estimated by the slope of the linear fit (red dashed lines).

DFA has been widely used to quantify the long-range correlation embedded in a non-stationary time series (Peng et al., 1995; Kiyono, 2015). The standard procedure of the DFA:

1. Observed time series {*x*_i_} (Figures 1A,D,G) after subtracting the mean from each data point (a) is integrated (blue solid lines in Figures 1B,E,H).
2. The integrated time series is divided into equal-sized, non-overlapping segments of length *n* samples (*n* = 200 in Figures 1B,E,H).
3. In each segment, the mean-square-deviation from the least-squares polynomial fit of degree *k* is calculated (*k* = 1 in Figure 1B). Depending on the polynomial degree *k*, the method is referred to as *k*th-order DFA or DFA*k*, in which non-stationary trends approximated by polynomial functions of degree (*k*-1) are removed from {*x*_i_}. The mean-square-deviations are then averaged over all segments and its square root *F*(*n*) is calculated. This computation steps (2) and (3) are repeated over multiple time scales (window sizes) to characterize the relationship between *F*(*n*) and *n* (Figure 1C). A linear relationship on a log-log plot of *F*(*n*) as a function of *n* indicates the power-law scaling range. In the scaling range, the fluctuations can be characterized by a scaling exponent α, the slope of the linear relation between log *F*(*n*) and log *n* (Figures 1C,F,I).

In conventional EEG spectral analysis, the mean characteristics of the power spectra averaged over extended periods of time have been studied to obtain statistically reliable characteristics. In that case, averaging procedures (resulting in a “static” picture) may mask the dynamical properties. To characterize such dynamical properties, we analyze the temporal fluctuations of EEG-band powers using DFA.

First, for each EEG channel, the spectrogram was obtained with the following parameters (window size = 10 s, window type = Hamming, 0.1 s overlap). Then, for each frequency band, the power spectrum density value time series was obtained. These sequences were then used as the input for performing the DFA.

**Figure 2:**
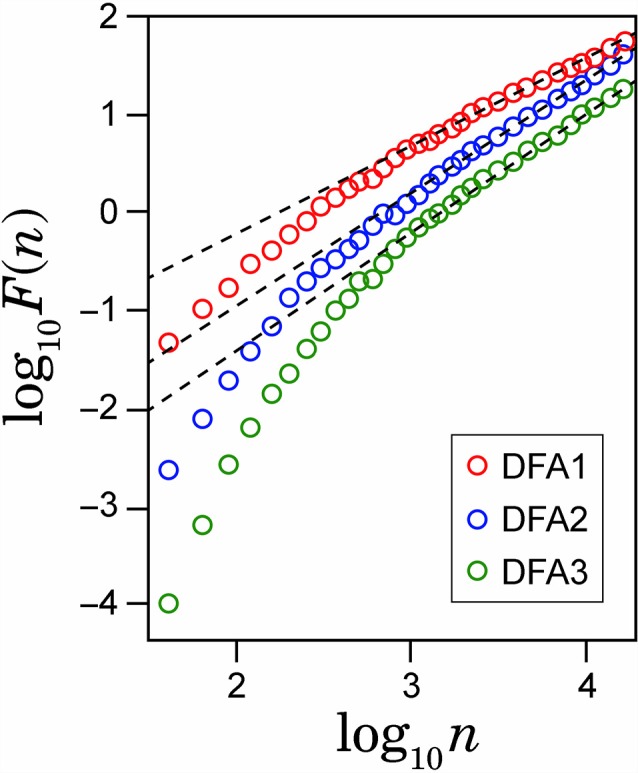
Example of the log-log plots for different orders of detrending polynomial (dotted line) and their respective linear approximations (solid line).

To check spurious detection of scaling behavior caused by a nonstationary trend embedded in the time series, we calculated double logarithmic plots for several orders of DFA. In Figure 2, the examples of the log-log plots for DFA1, DFA2 and DFA3 are presented with the straight lines (dashed lines) showing the linear approximations. The aim of this comparison among different orders of DFA is to evaluate the consistency of the estimated scaling exponent. As seen on the plots, after some point on horizontal axes, all plots for larger values of log(n) in different orders of DFA are converging to the same slope line of the log-log plots. The asymptotic slope convergence indicates that the correct scaling exponent can be estimated by higher-order DFA. Hence for this study, we used second order detrending for all feature time series.

In DFA applications, one often computes separately the scaling exponents for short-length window sizes *n*, and for long-length window sizes, describing the short-range and long-range persistence for the time series.

The reasoning behind the usage of DFA in our work is to employ its capability of identification long- and short-term correlations in the time series. We consider the correlation as a property of possessing similar characteristics over a certain time span. Depending on the duration of the period when the time series properties are stable, longer- or shorter-periods of the autocorrelation are observed.

As we aimed to find the locations when the neuronal activity is considerably stable, we need to use DFA to describe the long-range correlation of the human brain activity during the mental arithmetic task performance. We were interested particularly in the long-range scaling exponent. So, we developed the algorithm for automatic finding the crossover point between the short and the long-range scale in log-log plot. The second order detrending curve was analyzed and the position of the bending point was located based on the Akaike information criterion (AIC) between the fitted line with or without a crossover and the actual line. The point, where AIC value was the smallest one was the actual crossover point. After finding the crossover point, we used its coordinate as the bottom margin of log window sizes to calculate the scaling exponent α, the slope of the log-log plot (DFA scaling exponent). This parameter represents the autocorrelation properties of the time series:

1. *α* < 0.5 long-range anti-correlated signal
2. *α* = 0.5 uncorrelated signal (white noise)
3. *α* > 0.5 long-range correlated signal

### Power Spectral Density Maps

To compare our approach to the existing methods of quantification of brain activity during cognitive activation, we calculated the PSD based on Fourier Transform. This characteristic is a *de facto* standard measure of the strength of oscillatory activity which was widely used in the study of emotional activations (Davidson et al., 1990; Koelstra et al., 2012; Zhao et al., 2018). We calculated PSD for every EEG channel in every frequency range for every task. PSD was calculated by the Welch method (Welch, 1967; Marple and Marple, 1987) which is preferred for finite and imperfect signals under analysis. This method consists in averaging of modified periodograms, calculated by Fast Fourier Transform, which results in the reduction of the noise in the power spectrum. After obtaining the PSD in the corresponding frequency range, it was normalized to the maximum value for count and background, and then the PSD map was plotted over the head surface.

### Coherence

Another metric for comparison to DFA results is coherence. Coherence of the EEG activity in different brain regions is widely used to quantify the synchronicity of oscillations in two distinct areas of the brain (functional connectivity) for different conditions/states, such as working memory or mental disorders (Thatcher et al., 1986; Locatelli et al., 1998; Sauseng et al., 2005; Kropotov, 2010), emotions (Hinrichs and Machleidt, 1992; Aftanas and Golocheikine, 2001; Reiser et al., 2012). In the context of the present study, coherence is used as an indicator of the statistical similarity of the neural generators’ activity in a particular frequency range in two distinct areas of the brain. In this work, the coherence was calculated as follows.

For every frequency range, we took one of all possible pairs of different EEG channels and calculate the coherence (Bendat and Piersol, 2011) using the entire recordings. To assess the validity of the coherence between the current pair of EEG signals, the surrogate data analysis was performed (Faes et al., 2004). Surrogate signals were obtained using the phase randomization: for one of two EEG signals, the Fourier Transform was calculated, and a random number from the range −π…+π was added to the phase of each harmonic component. Then the inverse Fourier Transform was performed, and the coherence was calculated between one initial EEG and the surrogate EEG. This procedure of surrogate calculation was repeated 100 times for each pair of electrodes. Only the coherence which is significantly based on the *t*-test (*p* < 0.05) was considered valid and used for further analysis. In the result, we obtained the valid values of the EEG coherence between the pairs of the electrodes, for every frequency range and for two experiments: resting state and calculations (groups “B” and “G” separately).

### Statistical Analysis and Validation

With three sets of the calculated characteristics (PSD, coherence, and DFA scaling exponents) the statistical analysis was performed. All statistical tests were performed using the Python SciPy library. The general aim is to understand if the identifiable differences are present in the data from two groups of subjects during the mental cognitive workload. The null hypothesis was that there is no difference in the PSD, DFA scaling exponent, and coherence values between resting and counting states for both groups of subjects. To challenge this hypothesis separately for three characteristics separately, we assumed that the data are i.i.d. and as a first step check the distribution for normality using D’Agostino and Pearson’s tests. As a result, the distributions were not normal, therefore we applied the Wilcoxon rank test for paired samples to compare medians of the distributions.

For PSD values in every frequency range, the channels for which the median values of the characteristics were statistically different for resting and counting were identified. Then for these channels, the difference between medians for was calculated and its map across the head surface was analyzed. The statistical tests for DFA scaling exponent were conducted in the same way. If the median value of scaling exponent for counting is statistically different from that in rest state in some channel, it is used for further analysis. The confidence level of *p* = 0.05 was used as a threshold for both PSD and DFA medians comparison.

Then the statistical hypothesis testing was performed to define the pairs of the electrodes for which the coherence is different for resting state and counting state. To do this, the Wilcoxon test for difference of medians was performed for the data from all subjects in each group separately (*p* < 0.05). As all frequency bands (β1, β2, θ1, θ2) that were analyzed in this work are derived from the same EEG recording, Bonferroni correction was applied to account for the multiple comparisons. The 5% *p*-value was corrected to eliminate the multiple comparison problem by division by *N* = 4. Those coherences which differ between rest and count states were subdivided into three ranges: low (0.3 … 0.49), middle (0.5 … 0.69), and high (0.7 … 1.0) to analyze them separately.

Finally, in each group, the difference between medians was calculated and plotted in the graph with a red line connecting two electrode locations if coherence for counting is larger than during rest, and in blue otherwise. These graphs were analyzed separately for each range (high, middle and low), and for groups B and G.

## Results

**Figure 3:**
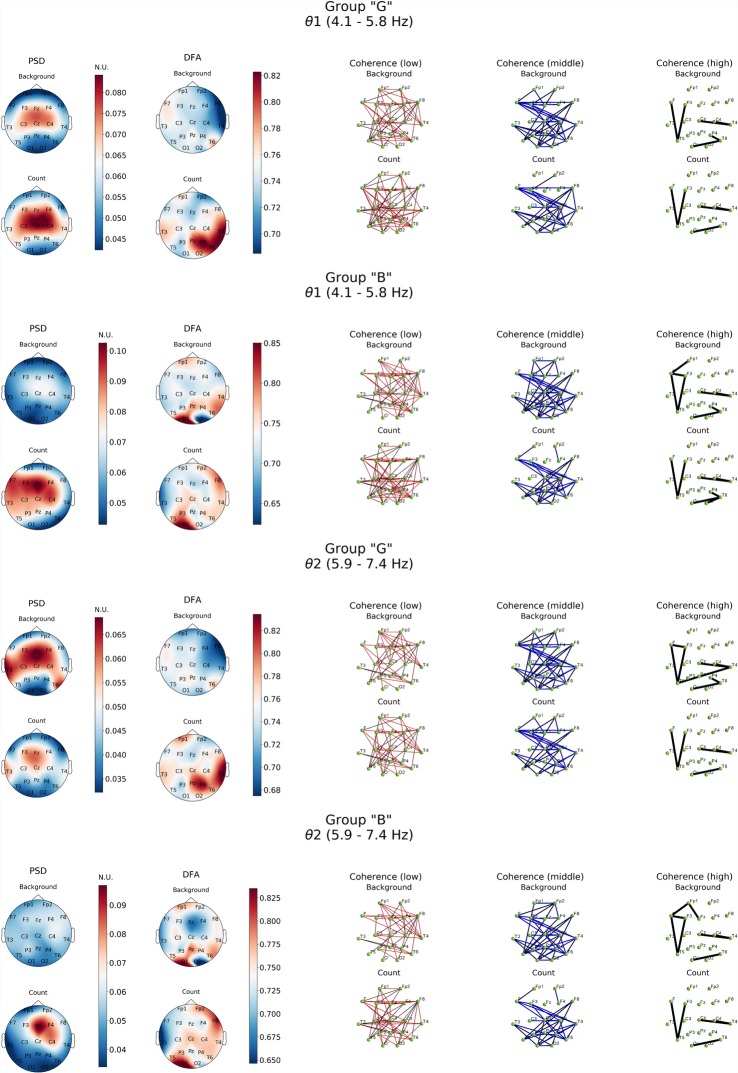
Topographic distributions of PSD, DFA exponent, and coherence in θ1 and θ2 electroencephalogram (EEG) subbands in groups with different evaluation of the task’s complexity (“G” and “B”) during resting state (Background) and mental calculations (Count). PSD, power spectral density; DFA, α values; Coherence, coherence coefficient value.

Analysis in both groups was aimed to describe differences in performing mental arithmetic task and subjective assessment of it (groups “G” and “B”) and neurodynamics of resting state for good and bad counters (Figure 3).

Resting state in group “G” was characterized by a distinct level of cortico-hippocampal networks activity and significant activation processes in both fronto-central regions, primarily expressed in θ2- subband, where exaltation of oscillations was already observed in temporal areas (T3, T6; Figure 3, PSD, Background). However, in group “B” the power of oscillations in both θ subbands during resting state was not expressed prominently.

**Figure 4:**
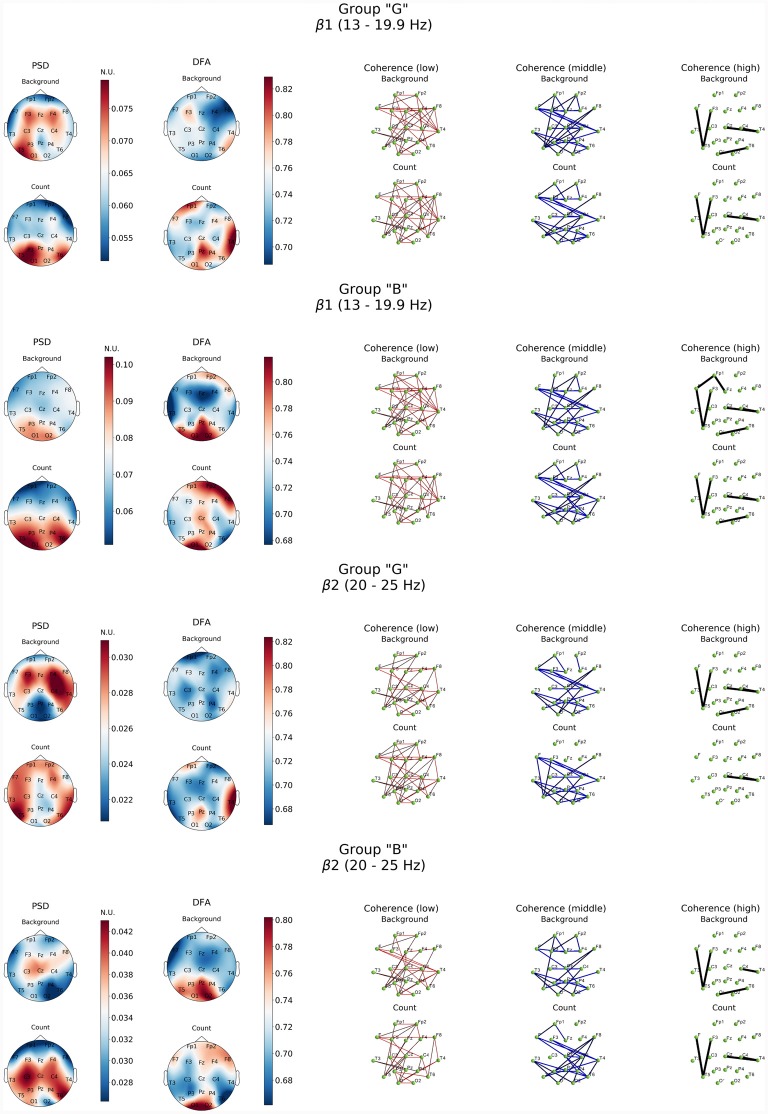
Topographic distribution of PSD, DFA exponent, and coherence in β1 and β2 frequency subbands in groups with different evaluation of the task’s complexity (“G” and “B”) during resting state (Background) and mental calculations (Count). PSD, power spectral density; DFA, scaling exponent values; Coherence, coherence coefficient value.

Similar patterns were observed also in high-frequency β-band dynamics during resting state (Figure 4). At the same time, “G” group was characterized by significantly higher power of β1 and β2 frequency subbands in both hemispheres of the brain with a prevalence of β1 in left frontal, posterior frontal and occipital cortical areas, alongside with generalized distribution across the cortex except for central occipital region. Prevalence of β2-subband oscillations was well expressed in the right hemisphere. In our opinion, certain topographic asymmetry of the activation processes identified within β-band fully corresponds to the functional role of the rhythms of these ranges. At the same time, power of high-frequency oscillations in “B” group can be explained by the formation of a pronounced β1-oscillations focus in left posterior parieto-temporal and both occipital regions and generalized distribution of β2-oscillations in central and right frontal cortical regions.

The processes associated with distant synchronization represented by coherence were rather similar in both theta- and beta-bands. No significant differences were observed in groups of subjects in terms of distribution topography. However, more well-defined functional connectivity distribution was revealed by a comparative analysis of DFA scaling exponent values in all EEG bands in both subject groups.

As it can be noticed from Figure 3, within θ subbands resting state in subjects of “G” group was described by the fact that values of DFA scaling exponent in all EEG bands were close to 0.5, which indicated the lack of long-range correlation in neural activity processes, particularly in right fronto-temporal cortical areas (medial area of right hemisphere cortex). Relatively similar pattern of changes in DFA scaling exponent was observed in this group in the range of β-band (Figure 4).

A completely different topography of DFA scaling exponent distribution was observed in the EEG recordings of “B” group. Resting state here was accompanied by a significant increase in temporally stable long-range correlation of EEG fluctuations (scaling exponent = 0.75, *p* < 0.05) in both θ subbands in temporal-parietal regions of the right hemisphere and left inferior frontal regions of the cortex. In the left occipital cortical area, DFA scaling exponent values reached 0.85 and higher (*p* < 0.05). Interestingly, in the right occipital area close to 0.5 DFA scaling exponent values were observed, which means the practical absence of temporally stable long-range correlation of EEG fluctuations (Figure 3).

Topography of the DFA scaling exponent values distribution within the range of β-oscillations in the “B” group was characterized by similarity in both subbands which consisted in a significant increase (up to 0.85) in the posterior regions of the neocortex (Pz, O1–O2, T5), alongside with mean values of DFA scaling exponent reaching 0.78 in frontal areas (Fp1, Fp2, F8) in β1-subband. However, DFA scaling exponent values in all other cortical areas did not exceed 0.7 (*p* < 0.05; Figure 4).

Generally close to 0.5 DFA scaling exponent values mean that the EEG fluctuations show uncorrelated behavior in all cases of our study of resting state (most prominently expressed in “G” group) may indicate the randomness of EEG dynamical characteristic.

Completely different EEG pattern was obtained during cognitive load. Execution of mental arithmetic task in subjects of “G” group was characterized by an increase in the magnitude of oscillatory power density of θ1-subband exclusively in orbito-frontal and central areas of either hemispheres and left temporal region (F7, T3, T5), while θ2-subband demonstrated a certain decrease in power. At the same time, in β1-frequency subband, formation of the PSD amplification center was observed in the posterior regions of the cortex (O1-T5-P3 and T6; *p* < 0.05; Figure 4).

**Figure 5:**
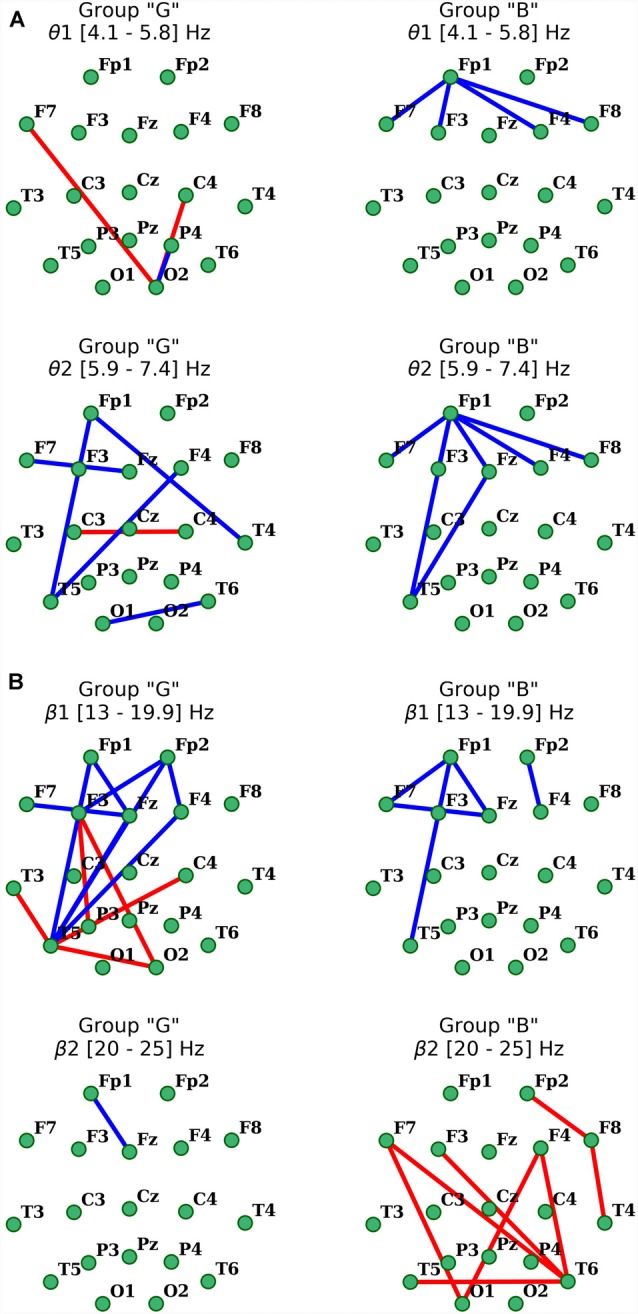
Distribution of changes in coherence in θ **(A)** and β **(B)** EEG bands during the execution of cognitive task compared to the initial resting state in groups with different subjective evaluation of the complexity of the task. Only statistically significant changes are indicated (*p* < 0.05), red indicates the increase, blue indicates the decrease.

Analysis of distant synchronization processes with coherence revealed that performance of a cognitive task in the participants of “G” group in the θ1-subbands was accompanied by formation of amplification focus with center in left temporal (T3) and right occipital areas (O2; Figure 5). However, within the range of θ2-subband, a significant decrease in functional interactions between frontal areas was observed, as well as a decrease in COH levels within frontal and F3, Fz–T5.

As for the β1-frequency oscillations, an increase of the functional association between cortical regions, such as the right occipital area parietal, temporal and frontal segments of both hemispheres was observed. Additionally, a significant decrease in the intrafrontal COH levels and functional connections of the left posterior temporal zone (T5) with frontal regions (Figure 5) were detected. In β2-subband only decrease in Fp1-Fz interaction was observed.

Furthermore, presence of active cognitive processes in “G” group was also revealed by DFA. Topography of the DFA scaling exponent values under cognitive activation within the range of θ1, 2-subbands was to a certain extent similar to each other. Likewise, similar patterns were observed for PSD results in the aforementioned subbands. Average values of scaling exponent were observed in Fp1, F4, F8 and T3, while maximum values were measured for Pz, P4, T4, T6 locations (scaling exponent > 0.8; *p* < 0.05). There were sustainably high (scaling exponent > 0.8; *p* < 0.05) and average (scaling exponent = 0.7; *p* < 0.05) values of DFA scaling exponent measured in left frontal, right parieto-temporal and right frontal areas within β1-subband, respectively (Figures 3, 4). However, β2-subband was characterized by the maximum regularity of oscillations only in the right temporal location (T4).

In the θ1-subband PSD (compared to the resting state), exaltation was observed with a dominance of the left hemispheric regions and central areas of the cortex. Within the range of θ2-subband, the focus of activation was expressed in the right central hemispheric regions (F4-C4-Fz; Figure 3). Wherein, the significant decrease of β1 frequency oscillations power (*p* < 0.05) was registered in the frontal areas. In the posterior areas (parietal-occipital; Figure 5), maximum values of power (*p* < 0.05) were recorded with a certain topographic dominance of the left-side cortical areas. In the topography of PSD distribution of the β2-subband against increased levels of activation processes were observed with maxima in C3 and T6, while minimum values were measured in the inferior frontal areas and right occipital zone (F7, Fp1-F8, Fp2, O2; Figure 4).

As for the results of DFA in “B” group, it turned out that in the θ1-subband cognitive task execution was accompanied by an increase of scaling exponent values in the frontal right-hemispheric (Fp2, F8) and central left-hemispheric (Cz, P3, T5) areas to the level >0.75 (*p* < 0.05), as well as a decrease in scaling exponent values in T3-F7-Fp1 locations to level <0.7 (*p* < 0.05). In the θ2-subband, a similar distribution of scaling exponent values was noted. However, a substantial decrease in the scaling exponent values to 0.65 (*p* < 0.05; Figure 3) was observed in T3-F7 locations. Analysis of β1-range oscillations revealed a change of occipito-frontal pole of dominance during resting state to the fronto-occipital one during cognitive activation (*p* < 0.05; Figure 4) with increasing scaling exponent values in the central areas (Fz-Cz-Pz, P4) as if manifesting interaction between the right frontal and left occipital regions of the cortex, which can be conditionally taken as indication of the intuitive cognitive axis emergence. Within the β2-subband, a decrease (compared to resting state) of the area of maximum scaling exponent values (>0.75) to occipital areas (O1–O2) was observed, alongside with increased scaling exponent values to the average level (≥0.75) in frontal areas (except Fp1, F3; *p* < 0.05; Figure 4).

Activation-related changes of cortical connections measured by coherence in “B” group also turned out to be quite different from those in “G” group (Figure 5). Performing a cognitive task in subjects of this group was accompanied by a prominent decrease in frontal distant synchronization processes in both θ subbands focused mainly in left prefrontal area, but more prominently in θ2 range oscillations, with foci in left anterior frontal (Fp1) and posterior temporal (T5) cortical areas (*p* < 0.05). Changes of COH for β-oscillations also had their own specifics in this group. Thus, the decrease in distant synchronization processes was observed only in the left fronto-temporal region. In contrast, changes of COH in “B” group, compared to “G” group, in the range of β2 oscillations had a significant increase of interhemispheric fronto-temporal functional connections, alongside with functional association in anterior regions of the right hemisphere (Fp2-F8-T4; *p* < 0.05; Figure 5).

Thus, from the results obtained it can be seen that subjective attitude to the performance of cognitive tasks (in our case, mental arithmetic task) has its own encephalographic manifestations, analysis of which may be helpful in understanding various brain mechanisms.

## Discussion

In our study, we focused on the analysis of θ- i β- frequency bands. EEG PSD as a parameter related to the strength of local synchronization processes was calculated alongside with detrended fluctuations analysis, which was used to quantify the stability of oscillations in time via long-term correlations. These characteristics were considered together with coherence analysis which estimates the synchronicity of the neural oscillations in the distinct parts of the brain.

First, we have to explain why the analysis of alpha frequency range was avoided in our study. Based on modern representations, α-activity (7.5–13.5 Hz) is associated with resting state, passive wakefulness, relaxation, etc. When its power increases, there is a decrease in the activity of the cortical zones observed alongside with increased activity in the thalamic zones. Its presence in the anterior areas of the neocortex is considered to evidence general low activation level of the brain.

At the same time, the views on the functional significance of α-oscillations have recently changed to some extent. So, according to Klimesch (1999), Röhm et al. (2001) and Sauseng et al. (2005), desynchronization in low- and mid-frequency sub-bands of the α-rhythm reflects activation of the thalamo-cortical loop and correlates with the processes of attention, while desynchronization in its high-frequency sub-band (mainly of cortical origin) reflects the enhancement of human cognitive activity.

Numerous sources have shown that the study of power fluctuations in the α-band of human EEG today is associated with processes of perception, attention, and memory. Consequently, the functional value of this EEG phenomenon remains not very well understood. Synchronization processes in α-band are nowadays taken to associate with suppression of mental tension and active inhibition of sensory information or irrelevant tasks.

In view of all of the aforementioned, we came to the conclusion that it would be inappropriate to include the analysis of data on changes in the α-band of EEG in this particular article because of the significant increase in the volume of the work and difficulty of its perception.

In the current study, we concentered our attention to a detailed analysis of the characteristics of beta and theta rhythms. Theta rhythm, generated in the limbic system, is considered to be “emotional” band of human brain, which refers to its role in cortical-limbic interactions. Hence it constitutes a cognitive component of emotional reaction and increase of its power cannot be simply reduced to activation transmitted from limbic system to different regions of neocortex (Demiralp and Başar, 1992). Moreover, according to modern concepts, an increase in theta-activity in the anterior cortical areas can be evaluated as a marker of its enhanced activation (Gundel and Wilson, 1992). Increased anterior frontal and midline theta synchronization, and enhanced theta long-distant connectivity between prefrontal and posterior association cortex with distinct “center of gravity” in the left prefrontal region (AF3 site) characterize states of internalized attention and positive emotional experience (Aftanas and Golocheikine, 2001). Thus, theta-activity in anterior-central regions of the human brain cortex is usually seen as basic, connected to cognitive functions implementation through cortico-hippocampal interactions and integrative in terms of functional connections between different cortical and subcortical structures (Başar et al., 2001a). Memory processes that are localized in the orbitofrontal cortex, are accompanied by an increase in the power of the θ-range of the EEG. In this case, the activation-cognitive view of the functional manifestation of θ-rhythms is confirmed by synchronization with β- and γ-oscillations and phase non-linear connection with the first ones (Schack et al., 2002).

There are two distinct functional θ-subbands, slow and fast cortical frequencies (Pastötter and Bäuml, 2014), which are differently related to the memory neural networks. The slow θ-oscillations are associated with the processes of remembering and awareness, and fast theta-subband reflects the emotional background of cognitive activity. It is well known that the magnitude of θ-oscillatory power density (specifically in θ1-subband) is significantly increased when complex mental problems are to be solved (Gundel and Wilson, 1992). Because of the widespread topography of shifts in θ-activity, it is now thought that left-hemispheric synchronization of oscillations in this band stands for formation and subsequent implementation of the analytical strategy of information perception, while activity in temporal, parietal and occipital cortical areas marks the initiation of extended cognitive analysis (Aftanas and Golosheykin, 2005).

At present time the beta range is mainly associated with various aspects of the functioning of the brain, from simple sensory reactions (visual, auditory, somatosensory, etc.) to higher cognitive functions, such as sensory memory, mechanisms of regulation of visual attention, movements, the processes of identification and cognition, emotional states and the implementation of cognitive, creative tasks (Özgören et al., 2005).

Activation processes in the β-range of human EEG are considered in connection with the analysis of various complex cognitive processes, memory mechanisms (recognition processes) and informational differentiation (Pulvermüller et al., 1997; Özgören et al., 2005), as a manifestation of activation mechanisms of cognitive control over behavioral reactions, attention focusing, estimation of stimulus significance (Cómez et al., 1998) and beginning of complex cognitive processes (Pulvermüller et al., 1997). Namely, oscillations in β-frequency band are associated with diverse functions and states, including the integration of incoming sensory information, selective attention, short-term memory, associative and perceptive learning. All of this provides evidence that synchronization in β-band is bound to such state of human brain as directed attention underlying cognitive abilities (Röhm et al., 2001; Rodriguez et al., 2004).

Furthermore, low-frequency β1-subband (13–20 Hz) is characterized by significant power increase when spatial visual attention processes take place, when β2-subband (20–30 Hz) is nowadays seen as a correlate of creative mental processes, heuristic task performing and verbalized thinking, as well as emotion regulation (Razoumnikova, 2000; Wróbel, 2000). Generally speaking, changes in β1-subband during the resting state today are seen along the lines of hypothesis, which assumes that oscillations of this particular frequency band maintain the status quo of current sensorimotor and cognitive state, in this means affecting the efficiency of cognitive control (Kukleta et al., 2003). On the other hand, the increase observed in β2-subband is thought to reflect synchronization of different polymodal information processing mechanisms during active thinking. This EEG-activity is linked to higher cognitive functions and noesis *per se*, videlicet defining the essence of the phenomena (Greicius et al., 2009).

Recently it has been shown, that both slow and fast cortical theta oscillations are critically involved in human episodic memory retrieval, being related to processes of recollection and conscious awareness, and fast theta oscillations being linked to processes of interference and interference resolution (Pastötter and Bäuml, 2014). Given the assumption that functional link between increased θ-activity in anterior and posterior cortical areas and cognitive/emotional processes depicts enhanced activation related to cortico-limbic interactions (Başar et al., 2001b), it can be presumed that execution of cognitive task will induce various changes in activation values of the corresponding neural networks of the brain and that should correlate with subjective assessment of complexity of the task performed. The concept of β-band as a marker of complex cognitive processes, memory mechanisms (i.e., recognition) and information processing (Pulvermüller et al., 1997; Schneider et al., 2016) also speaks in favor of this hypothesis. Furthermore, values of θ/β ratio are increasingly used these days as a marker of arousal or cognitive processing capacity (Clarke et al., 2019). EEG bands and cortical regions mentioned above are seen as those carrying cognitive functions and reflecting initiation of memory networks, attention and verbalization mechanisms (Klimesch, 1999; Razoumnikova, 2000; Fuster, 2015; Haber, 2016; Näätänen, 2018). A noteworthy feature of EEG-recordings in “B” group during the execution of a cognitive task lied in a completely different neurodynamics observed.

According to our results, the analysis of the brain functional connectivity and oscillatory power of the human cortical bands revealed functional structure of human brain activity both during resting state (activity of default mode networks and resting state neural networks, which provide the basis for internal mental activity (Karapanagiotidis et al., 2018) and during the execution of an intensive cognitive task (arithmetic task), confirming functional predictive values of θ and β frequency bands (Sauseng et al., 2010; Fries, 2015; Schneider et al., 2016). It is evident that initial resting state in the groups of participants was accompanied by the activation of mechanisms reflecting internal and external stimuli (self-assessment, thinking), that is, stream of consciousness, which includes activation of episodic memory, internal speech, mental representation, imagination, emotions et cetera (Greicius et al., 2003, 2009). However, it is quite possible to assume that in “G” group, these processes were much more powerful and state of conscious rest in these subjects was more closely connected with mind-wandering—the free flow of thoughts (Mason et al., 2007). This, in essence, reflects the unfocusedness of informational cognitive component of this state of brain activity. It is well known that cognitive processes are associated with the resting state (spontaneous cognitive processes, personal thoughts etc.; Buckner et al., 2008; Hasson et al., 2009). Therefore, in our case, the DFA allows describing additional features of the ongoing mental activity of the resting brain.

It is worth noting that major changes in both powers of oscillations and levels of coherence had localization specificity for the state being analyzed and were reasonably specific for coherence values (formation of specific foci in coherence topography). At the same time, visualization of changes in coherence turned out to be the most representative, which in turn gave a clear neurophysiological basis for the difference in brain mechanisms of information processing in our experimental groups (Figure 5). Thus, our results confirm the presence of well-established EEG correlates of the effect of subjective task complexity evaluation on elementary cognitive task performance as described in Schmidt et al. (2018).

DFA of activation rearrangement in EEG dynamics suggests that normal functioning of the brain is characterized by long-term temporal correlations between regions of the cortex, thereby supporting decision-making processes and memory mechanisms (performing cognitive tasks). At the same time, studies of the initial resting state show that the resting brain works on the threshold of dynamic instability, which is expressed in a low scale of time correlations in the vast majority of cortical areas, regardless of the typology of emotional response to cognitive activity. In this, our data is to a certain extent synergistic with the results of Daffertshofer et al. (2018). However, it should be noted that in “B” group during resting state high scaling exponent values were observed in the posterior and partially anterior regions of the neocortex in both θ and β-bands, which differed from the EEG data from “G” group (Figures 3, 4). Therewith, at present, the posterior areas of the cortex are associated with neural networks that are actively involved in the implementation of cognitive reactions (working memory), in particular in control systems (Schiffler et al., 2016).

Topographical distributions of the DFA scaling exponent were comparable for θ and β frequency bands, demonstrating the largest values of DFA scaling exponent during cognitive activation in both groups of subjects. The long-term temporal correlations were stronger in the “G” group especially in θ-band and involved mainly right temporal, parietal and occipital regions, which might be related to additional strengthening of the mechanisms of verbal memory (Shinoura et al., 2011). Increase in the DFA scaling exponent values in β-band and coherence values in β2-subband in the “B” group was topographically related to the anterior and posterior areas—the ones that are actively involved in the implementation of cognitive activity, including regions displaying somatic background of emotional response. In this regard, it is possible to assume that it is due to the increased level of cognitive activity, including systems of downward control (both during resting state and cognitive task performing), that difficulties and negative emotions elicited by cognitive task were encountered.

Obtained values of DFA exponent suggest that normal functioning of the brain is characterized by long-term temporal correlations in the cortex, which are involved in decision-making and memory. At the same time, it is important to note that according to our results, presence or absence of long-range temporal correlations may represent the greatest interest for diagnosis of the variety of brain dysfunctions of different etiology, including emotional and cognitive impairments. In addition, its use has enabled us to integrate more subtle mechanisms of working memory activation (in regions of right hemisphere), which cannot be achieved, for example, by means of PSD analysis. Furthermore, the results obtained by DFA coincide quite well with the results of fMRI studies (Zago et al., 2008).

We assume that DFA may also be an useful tool in the determination of psycho-neurological status and for control of pharmacological treatment efficacy of pathologies that are accompanied by stable tonic states (e.g., phobias), informational exhaustion and psycho-emotional overload, including post-traumatic stress disorder. The important data in support of this assumption were obtained on the use of DFA in the study and treatment of neurological diseases. The strength of long-term temporal correlations was significantly lower in patients with infantile spasms (West syndrome) than in control patients, indicating a decrease in the control of neural synchrony (Smith et al., 2017). There were no differences between patients without hypsarrhythmia and control patients. In addition, the presence of hypsarrhythmia can be classified based on the results of DFA. Successful treatment was marked by an increase in DFA scaling exponent values compared to data obtained from patients with persistent spastic activity. These results suggest that the strength of long-range time correlations is a marker of pathological cortical activity that correlates with response to treatment. In combination with current clinical measures, this quantitative tool can help to objectively identify hypsarrhythmia and evaluate the effectiveness of treatment to inform patient about clinical decision making.

Furthermore, the recent introduction of DFA technique for heart rate variability measuring appears to provide improved prognostic methods of cardiovascular diseases diagnostics by calculating the alpha fractal scaling component (Willson et al., 2002; Chiang et al., 2016). As it was noted by Arsac and Deschodt-Arsac (2018), in the last decade there has been a growing interest in fractal physiology among neuroscientists and clinicians. Many physiological systems coordinate themselves to reduce variability and maintain a steady-state. Further advancement of application of DFA to studying of brain behavior is in focus of our research.

## Conclusion

DFA proved to be efficient in terms of brain functional state estimation, as it reflects long-term correlations of oscillatory processes and can provide information about the reliability of cognitive engagement or initiation of certain brain functions during formation of behavioral strategies with noticeable activation component.

Based on the analysis of EEG recordings obtained during mental arithmetic task performance (serial subtraction), we demonstrated the ability of the DFA to showcase the long- and short-term connections in EEG time series, related to the changes in EEG dynamics due to the cognitive workload. Current study provides evidence that DFA used jointly with PSD and coherence, shows benefits of DFA application to describe durable informational connections in the process of human cognitive activity reveals comprehensive information about strength, duration and spatial connectivity of neuronal oscillations, which can advance our knowledge about processes in the brain under various conditions. However, it should still be stressed that the physiological value of the scaling exponent parameter of DFA remains to some extent unclear and requires its further detailed neurophysiological study.

## Data Availability

The dataset analyzed for this study can be found in the Physiobank repository: https://physionet.org/physiobank/database/eegmat/.

## Ethics Statement

Bioethics Commission of Educational and Scientific Center “Institute of Biology and Medicine” of Taras Shevchenko National University of Kyiv examined this work. All experiments were designed in accordance with Helsinki Declaration (International Medical Assemble), international principles of the European Convention for the protection of vertebrate animals were used for experimental and other scientific purposes (European Convention, Strasburg, 1986), Declaration of Principles on Tolerance (28 sessions of UNESCO, Paris, 1995), Universal Declaration on Bioethics and Human Rights (UN, 1997), norms of the Convention for the Protection of Human Rights in the field of new biomedical technologies adopted in 1997 in the city and article 26 of the Law of Ukraine “On protection of animals from cruelty” (No 3447-IV, 21.02.2002). All experiments are considered as in accordance with the norms of bioethics, following international regulations for the conduct of experimental work and clinical trials.

## Author Contributions

IZ, AP, KK and IS contributed to the conception, methodology and design of the study. ST and IZ designed and organized the data collection. OS and IS organized the database. IS, AP and KK curated the data. IS and AP created the software. IZ, KK and AP provided validation and formal analysis. IS conducted statistical analysis and visualization. IS, IZ, ST, KK, AP, OS and MC prepared the original draft of the manuscript. AP administered the project. All authors contributed to manuscript revision, read and approved the submitted version.

## Conflict of Interest Statement

AP and IS were employed by company Ciklum. The remaining authors declare that the research was conducted in the absence of any commercial or financial relationships that could be construed as a potential conflict of interest.
